# Microbial consortia and their metabolic modulations as mechanisms of water deficit tolerance in strawberry plants

**DOI:** 10.3389/fpls.2026.1843067

**Published:** 2026-07-07

**Authors:** Urley A. Pérez-Moncada, Luis Bustamante, Cledir Santos, Ricardo Cabeza, Paula Pimentel, Pablo Cornejo

**Affiliations:** 1 Doctorado en Ciencias de Recursos Naturales, Universidad de La Frontera, Temuco, Chile; 2 Laboratorio de Microbiología Agrícola, Centro de Investigación Tibaitatá, Corporación Colombiana de Investigación Agropecuaria (AGROSAVIA), Mosquera, Colombia; 3 Doctorado en Ciencias y Tecnología Analítica, Universidad de Concepción, Concepción, Chile; 4 Departamento de Análisis Instrumental, Facultad de Farmacia, Universidad de Concepción, Concepción, Chile; 5 Núcleo Científico y Tecnológico en Biorecursos BIOREN-UFRO, Universidad de La Frontera, Temuco, Chile; 6 Postgraduate Program in Biotechnology, Federal University of Technology- Paraná, Ponta Grossa, Brazil; 7 Centro Tecnológico de Suelos y Cultivos (CTSyC), Facultad de Ciencias Agrarias, Universidad de Talca, Talca, Chile; 8 Departamento de Fisiología Vegetal, Centro de Estudios Avanzados en Fruticultura (CEAF), Rengo, Chile

**Keywords:** arbuscular mycorrhizal fungi, bacteria, drought stress, microbial biostimulants, nutrients, yeast

## Abstract

**Introduction:**

One effective and ecologically sustainable strategy that has emerged to enhance plant resilience to water deficits induced by drought stress is the application of plant growth-promoting microorganisms (PGPM). Despite this progress, there remains a lack of clarity in identifying the most effective microbial consortia and understanding the nutritional and metabolic processes that contribute to the drought resilience of strawberry crops.

**Methods:**

Two effective microbial consortia were selected based on previous data: CS1 (*Claroideoglomus claroideum* + *Naganishia albida* + *Paraburkholderia caledonica*) and CS2 (*Funneliformis mosseae* + *Candida guillermondii* + *Bacillus tequilensis*) used for drought stress mitigation in plants. These were tested under two irrigation scenarios–well-watered and water-limited conditions, corresponding to 85% and 30% of their water holding capacity (WHC), respectively.

**Results:**

The co-inoculation of the CS1 consortium increased SDW by 38.23% compared to the control and 52.94% compared to CS2. Likewise, this consortium increased the concentrations of N, P, and K by 24.3%, 6.4%, and 25.7%, respectively, compared to the WS control. Finally, CS1 mitigated phytotoxic effects by activating flavonoid-type, fatty acids, and phenolic acid metabolites, as well as growth-regulating hormones, compared to the CS2 consortium and control plants, whose changes in specialized metabolism did not allow them to tolerate stress.

**Discussion:**

This study elucidated the molecular mechanisms by which microbial biostimulants increase drought tolerance.

## 1 Introduction

Biotic and abiotic stressors, including pathogen attacks, soil salinity, heavy metal contamination, elevated temperatures, and drought conditions, substantially affect agricultural productivity, thereby influencing global food security (Yang et al., 2023; Aina et al., 2024). In the context of drought stress, insufficient soil moisture leads to a water deficit within plant cells, resulting in significant alterations in physiological, biochemical, and metabolic processes. This is manifested as a reduction in agricultural yield, with potential losses ranging from 10% to 90% (Hussain et al., 2019; Sati et al., 2022; Khan et al., 2025). For instance, water deficit in strawberry plants can reduce total fruit production by approximately 80%, accelerate ripening, and substantially decrease fruit size and yield (Adak et al., 2018; Zahedi et al., 2023; Yenni et al., 2022; Pérez-Moncada et al., 2024). In response to a range of harmful external factors, plants can initiate defense mechanisms through various modulation pathways. These include stomatal reactions, osmotic regulation, buildup of osmolytes, and activation of both enzymatic and non-enzymatic antioxidant defense systems. Additionally, they can adjust phytohormones, signaling genes, transcription factors, and reconfigure their metabolome (Aina et al., 2024; Haghpanah et al., 2024). However, ensuring safe and sustainable global food production to feed an ever-growing population remains a challenge in a world where this is a priority (Aina et al., 2024; Khan et al., 2025). One effective and ecologically sustainable strategy that has emerged to enhance plant resilience to water deficits induced by drought stress is the application of microbial biostimulants (MB), which can be inoculated as either individual strains or consortia. The use of MB in strawberry plants has achieved significant advances that are crucial for improving plant growth under water deficit conditions and enhancing the mechanisms that plants use to cope with drought (Morales-Quintana et al., 2022; Paliwoda et al., 2022; Aina et al., 2024; Yanez et al., 2025; Shirdel et al., 2025). MB enhance plant health by facilitating nutrient uptake (Das et al., 2022; Sharma et al., 2025) and modulating primary, secondary, or specialized metabolic processes (Kaur and Suseela, 2020; Lin et al., 2022; Sharma et al., 2025). Secondary metabolism plays a crucial role in enabling plants to endure stressful biotic or abiotic conditions; however, unlike primary metabolism, it does not constitute a component of normal plant development (Al-Khayri et al., 2023).

Historically, individual strains have been extensively used as biotechnological tools to enhance plant resistance to various environmental stresses. Nonetheless, the contemporary focus has shifted to the development of microbial consortia to improve stress tolerance in plants (Negi et al., 2024). It is hypothesized that the use of microbial consortia provides a broader spectrum of ecological functions than single-strain inoculants and enhances stability across diverse environmental conditions. Although single strains have proven effective, consortia may offer multi-trophic interactions that remain unexplored in crops (Nunes et al., 2024; Negi et al., 2024). Under stress conditions, such as water deficit, the application of microbial consortia to various plant species has been demonstrated to enhance tolerance to stress by remodeling and activating diverse compounds within the specialized metabolism of plants (Lin et al., 2022; Abou Jaoudé et al., 2025a). For instance, Abou Jaoudé et al. (2025a) evaluated a synthetic microbial consortium (SynCom) consisting of five gram-negative bacteria known for their ability to solubilize phosphate, produce auxin, and/or fix nitrogen to assess its impact on enhancing plant growth and drought resistance in *Salvia officinalis*. The authors observed that SynCom, when applied under well-watered conditions, induced metabolic responses like those typically observed under water stress. This finding suggests that inoculation may initiate a drought-like acclimation process, potentially involving mechanisms such as stomatal regulation and the mitigation of oxidative stress. The alteration of specific metabolites, including histamine, α-ketoglutaric acid, and anserine, appears to play a role in these processes (Abou Jaoudé et al., 2025a). Conversely, the observed increases of 183.83% and 164.97% in moderate and severe drought conditions for Astragaloside IV, and the increases of 86.60%, 148.56%, and 111.45% in mild, moderate, and severe drought stress conditions, respectively, for the flavonoid calycosin-7-glucoside in *Astragalus mongholicus* plants inoculated with bacterial consortia, provide novel insights into enhancing drought stress responses. These findings also contribute to the accumulation of medicinal compounds used in traditional Chinese medicine (Lin et al., 2022). Although the use of microbial consortia to enhance plant growth, disease control, insect pest control, and plant tolerance to other environmental stresses is a current trend, there is still limited information on their use in improving water stress tolerance. To date, the interactions between strawberry plant-microbial consortia and the plant metabolome’s response to water stress have not been investigated. Additionally, the precise mechanisms through which these consortia affect plant metabolic reprogramming under water stress conditions remain incompletely understood. It is crucial to comprehend the mechanisms governing the interactions between plants and microbial consortia and develop strategies that enhance crop resistance to water scarcity by improving productivity under changing environmental conditions. Therefore, this study aimed to assess the impact of inoculating plants with two microbial biostimulants, each comprising three groups of microorganisms: arbuscular mycorrhizal fungi (AMF), plant growth-promoting bacteria (PGPB), and yeasts. The focus was on examining the metabolic responses of plants under water stress conditions, with particular attention to identifying the specialized metabolic processes and biochemical pathways that contribute to stress tolerance. These findings are expected to provide novel insights into the role of microbial consortia in enhancing the resilience of strawberry plants in environments with limited water availability. This research may lead to the development of targeted microbial inoculants as a biotechnological tool that must be validated under field conditions without compromising food safety.

## 2 Materials and methods

### 2.1 Plant material, and microbial biostimulants selection

An experiment was conducted to evaluate changes in foliar secondary metabolism of strawberry plants cv ‘Alexandria’ inoculated with two microbial biostimulants growing under water deficit. The experiment was carried out in the greenhouses of the Departamento de Ciencias Químicas y Recursos Naturales, Universidad de La Frontera, Temuco, Chile. Strawberry plants were obtained from surface-sterilized seeds grown in a sterile substrate consisting of peat and perlite (1:1, v/v). Seed surface disinfection was performed using a sodium hypochlorite (NaClO) solution for five min, followed by three rinses with sterile distilled water under a laminar-flow cabinet. Seeds were then placed in Petri dishes and incubated at 25 °C ± 2 until germination. After germination, seedlings were transferred to germination trays containing sterile peat:perlite (1:1, v/v). After two months, plants were transplanted into 1 kg pots filled with autoclaved soil (121 °C, 15 psi, 2 h, one cycle).

Two microbial consortia were selected based on previous data obtained (Pérez-Moncada et al., 2024): CS1 (*Claroideoglomus claroideum* + *Naganishia albida* + *Paraburkholderia caledonica*) and CS2 (*Funneliformis mosseae* + *Candida guillermondii* + *Bacillus tequilensis*). The evaluation involved three treatments (CS1, CS2, and uninoculated control (CT)) and two irrigation conditions (well-watered and water-deficient plants, at 85% and 30% of their water holding capacity (WHC), respectively) under a completely randomized design. The water deficit began on 31st day, after the plants were transplanted into 1 kg pots (the plants reached a growth stage of 91 days). There were five experimental units per treatment, for a total of 30. Inoculation of the consortia was performed three times: (i) after plant germination (25 days after germination), (ii) at the time of transplanting into 1 kg pots containing sterile soil (60 days after germination), and (iii) at the onset of water stress (31 days after the transplant). Arbuscular mycorrhizal fungi were inoculated only once at the beginning of the experiment, at a rate of 100 spores per plant. Bacterial and yeast inoculations were applied directly into the substrate at concentrations of 10^9^ and 10^6^ colony-forming units (CFU) mL^-^¹, respectively, at all three inoculation times. Each plant received 10 mL of the mixture (5 mL of bacteria and 5 mL of yeast). The plants were harvested 90 days after the water deficit was imposed for analysis of above-ground and root biomass, nutrient concentration and metabolite extraction from the leaves.

### 2.2 Plant growth and arbuscular mycorrhizal fungi traits

Shoot and root dry weights (SDW and RDW, respectively) were determined by drying each organ in an oven at 70 °C for 72 h. The relative water content (RWC) was measured according to the method described by Aroca et al. (2003). Root colonization by AMF was quantified at 30 days after the hydric stress (DAS) and at the end of the experiment (90 DAS), using a dissecting microscope (20-40X) after treating a portion of roots in KOH solution and staining with 0.05% (w/v) trypan blue in lactic acid (Phillips and Hayman, 1970). Measures of root colonization were taken using the method described by Trouvelot et al. (1986).

### 2.3 Nutrient concentration in strawberry leaves

Strawberry leaves dried in an oven at 70 °C for 24 h and subsequently ground and sieved on a 0.5 mm diameter sieve were used for the determination of nutrients such as nitrogen (N), phosphorus (P), potassium (K), calcium (Ca), and magnesium (Mg). The quantification of P was performed by the colorimetric method after the formation of blue molybdate (Sadzawka et al., 2007); meanwhile, the N concentration was obtained by using the colorimetric method (Sadzawka et al., 2007). K, Ca and Mg content was determined by Atomic Emission Spectroscopy with Inductively Coupled Plasma (ICP-OES) after acid digestion.

### 2.4 Extraction of samples for secondary metabolite analysis from strawberry leaves

Secondary metabolites were extracted from freeze-dried, ground strawberry leaves. Four compound leaves were collected from each treatment group, with three replicates per treatment, for further analysis. Two extraction techniques were employed to encompass most of the secondary metabolites within the plant: i) Biphasic extraction to cover polar metabolites using the method described by Matyash et al. (2008), with slight modifications (Pérez-Moncada et al., 2026). For polar compounds, profile analysis 500 µL of the supernatant was recovered in a new 2 mL tube. The samples were dried in a SpeedVac overnight. The samples were reconstituted with 200 µL of cold acetonitrile (ACN)/H_2_O (80/20), mixed, and subjected to ultrasound for 5 min. The samples were centrifuged at 10,000 *g* for 10 min, and an aliquot of the supernatant was transferred to vials with a glass insert for subsequent analysis by Hydrophilic Interaction Liquid Chromatography (HILIC) HILIC-MS/MS; and ii) Single-phase extraction to cover metabolites of medium polarity. In brief, a portion of 60 mg of sample was mixed with 1,000 µL of cold MeOH/H_2_O (80/20 v/v). The samples were then mixed in a ThermoMixer shaker at 2,000 rpm for 3 min at 4 °C. After, the samples were centrifuged at 15,700 *g* for 10 min at 4 °C and subsequently, 800 µL of the supernatant was recovered in a new 2 mL tube. The samples were dried in a SpeedVac for 4 h at 40°C. Finally, the samples were resuspended with 750 µL of MeOH/H_2_O + 0.1% formic acid (FA) and 100 µL were taken and transferred to analysis by UHPLC-MS/MS.

### 2.5 Analytical conditions for UHPLC-MS and its HILIC-MS variant

Procedure was carried out with a UHPLC-DAD Bruker Elute LC system coupled in tandem with a Q-TOF spectrometer Compact, Bruker (Bremen, Germany) coupled to an electrospray ionization (ESI) source. The control system used was Compass HyStar (Bruker), and the acquisition software was Bruker to control 4.1.402.322-7977-vc110 6.3.3.11.

For the chromatographic separation of the medium polarity metabolites was carried out using a Phenomenex Kinete C18 column (100 x 4.6 mm, 2.6 µm) (Torrance, CA, USA). The MS conditions in metabolomics studies were negative ionization ESI(-) 4,500 V; at a flux of 0.6 mL/min; capilar T° of 200 °C; collision energy at 10–40 eV; Auto MS/MS mode (2 precursor/cycle), 50–1,500 *m*/*z* (scan 0.2 s centroid mode); and internal calibration using sodium formate (0.01 M) with a mass accuracy < 3 ppm. The mobile phases used, consisted of (A) formic acid 0,1%, ammonium formate 10 mM, in 60:40 acetonitrile – AcN/H_2_O and (B) formic acid 0,1%, ammonium formate 10 mM, in 90:10 IPA/AcN.

On the other hand, for chromatographic separation of the polar compounds was carried out using a Luna NH2 column (Phenomenex) with dimensions of 2 mm × 100 mm and a particle size of 3 µm. The column was maintained at a temperature of 40 °C and the samples were kept at a temperature of 8 °C with an injection volume of 3 µL per sample. The polar compounds were analyzed in both positive and negative ion modes, with a capillary voltage of 4,500V for ESI(+) and 3,000V for ESI(-), and an end-plate offset of -500V. The nebulizer gas was set at 1.8 Bar with a dry gas flow rate of 9 L/min and temperature at 230 °C, with a mass range of 50 to 1,300 *m*/*z*. The mobile phase used, consisted of (A) water + 0.1% acetic acid, 10mM ammonium acetate and (B) 99% acetonitrile + 0.1% acetic acid + 10mM ammonium acetate.

### 2.6 Statistical analysis

The data obtained in CSV format were analyzed using MetaboAnalyst 6.0 software (Pang et al., 2024; https://www.metaboanalyst.ca/). All data from the different treatments and the two irrigation conditions were normalized by the median, log transformers and scaled using Pareto scaling method and clustered into a heat map. Furthermore, Partial least squares discriminant analysis (PLS-DA), a form of multivariate analysis, was conducted to examine the classification of data sample groups and assess the differences among these groups. Following this, a univariate analysis was performed in order to identify significant secondary metabolites using the following criteria: *p <* 0.05 in the Student’s T-test, and log_2_FC < − 2 or log_2_FC > 2 and plotted in a Volcano plot. Significant metabolites were annotated using Metaboscape 4.0, MS-FINDER v. 3.61 and SIRUS v. 6.0.6. In addition, for shoot and root dry weights, the relative water content (RWC), root colonization by AMF, and nutrient concentration in strawberry leaves, two-way ANOVA were performed. Kolmogorov-Smirnov and Shapiro-Wilk test were used to check the normality and homogeneity of the data, respectively. Significance in the means of the data was separated using Tukey’s test at the 0.05 probability level using R software v. 4.3.0.

## 3 Results

### 3.1 Plant growth and arbuscular mycorrhizal fungi traits

Shoot dry weight (SDW) and root dry weight (RDW) were strongly influenced by inoculation type (IN) and irrigation condition (IC) (*p ≤* 0.05) (**Table 1**). Water deficit significantly decreased shoot dry weight (SDW) and root dry weight (RDW) by 57.14% and 24.32%, respectively, in the water stressed (WS) control, compared to the well-watered (WW) control (**Table 1**). Plants inoculated with CS1 (*C. claroideum* + *N. albida* + *P. caledonica*) had a greater effect on SDW than the control and CS2 under the two irrigation conditions studied. Under WW conditions, CS1 increased SDW by 21% and 25.80% compared to the control and CS2, respectively. Whereas, under WS conditions, CS1 increased SDW by 38.23% compared to the control and by 52.94% compared to CS2. In contrast, under the two irrigated conditions, CS2 had no effect on SDW production in strawberry plants compared to their respective controls (**Table 1**). On the other hand, although no statistically significant influence of inoculation type, irrigation condition and interaction between these factors on RWC was observed, an increase in the values of this variable in stressed CS1 (94.9 ± 2.65%) compared to control (82.6 ± 6.67%) and stressed CS2 (79.5 ± 2.17%) treatments was registered.

**Table 1:** Shoot (SDW) and root dry weight (RDW), and relative water content (RWC) in strawberry plants inoculated with microbial consortia under non-stress condition (WW) and under water deficit (WS) conditions.

Treatments	Irrigation condition	SDW (g)	RDW (g)	RWC (%)
Control	WW	4.9 ± 0.26b	3.7 ± 0.06b	90.0 ± 3.45ab
CS1	WW	6.2 ± 0.52a	5.2 ± 0.32a	78.6 ± 5.51b
CS2	WW	4.6 ± 0.34b	2.7 ± 0.45c	81.8 ± 4.81ab
Control	WS	2.1 ± 0.36d	2.8 ± 0.35c	82.6 ± 6.67ab
CS1	WS	3.4 ± 0.26c	2.5 ± 0.12c	94.9 ± 2.65a
CS2	WS	1.6 ± 0.24d	2.2 ± 0.23c	79.5 ± 2.17b
Significance				
IN		***	**	ns
IC		***	***	ns
INxIC		ns	**	ns

WW, well-watered, irrigated up to 85% water holding capacity (WHC); WS, water-stressed, irrigated up to 30% WHC. CS1, *C. claroideum* + *N. albida* + *P. caledonica*; CS2, *F. mosseae* + *C. guillermondii* + *B. tequilensis*; IN, inoculation type; IC, irrigation condition. The significant difference was depicted as ns, not significant; *p < 0.05, **p < 0.01, and ***p < 0.001. Values represent means ± SE. Different letters indicate significant differences using Tukey’s test (*p ≤* 0.05).

**Figure 1:**
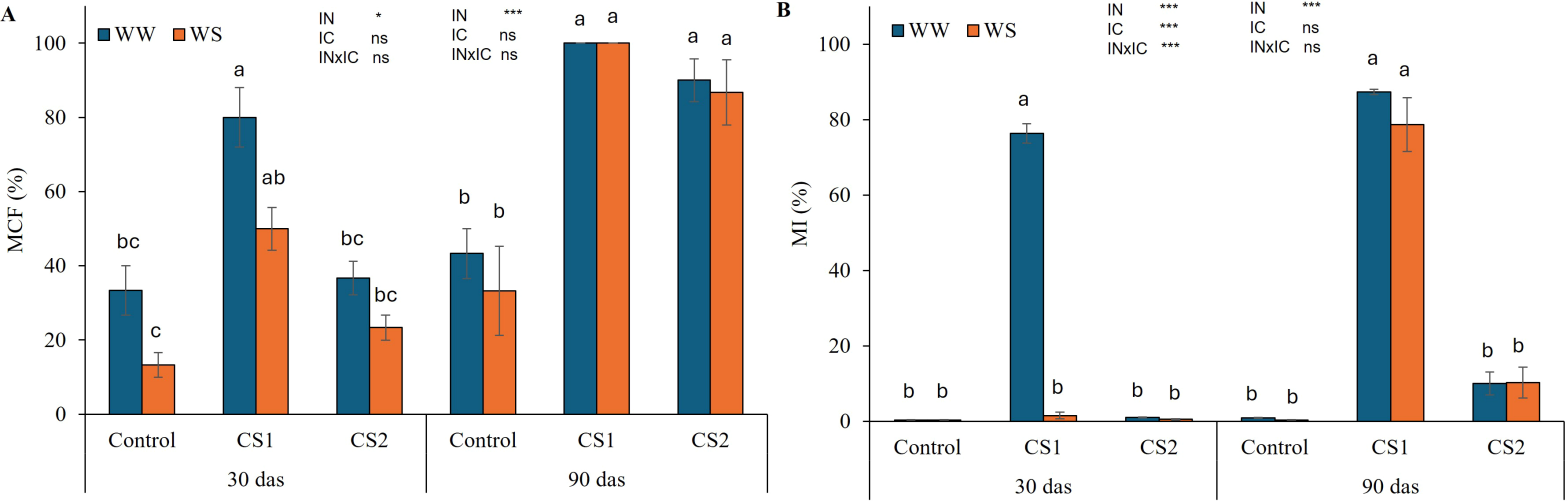
Mycorrhizal colonization frequency (MCF) **(A)**, and mycorrhization intensity (MI) **(B)** in strawberry plants inoculated with microbial consortia under non-stress condition (WW, dark blue) and under water deficit (WS, orange) conditions at 30 y 90 DAS. CS1: *C. claroideum* + *N. albida* + *P. caledonica*; CS2: *F. mossea* + *C. guillermondii* + *B. tequilensis*; IN, inoculation type; IC, irrigation condition. The significant difference was depicted as ns, not significant; *p < 0.05, **p < 0.01, and ***p < 0.001. Values represent means ± SE. Different letters indicate significant differences using Tukey’s test (*p ≤* 0.05).

The mycorrhizal colonization frequency (%MCF) and mycorrhization intensity (%MI) were influenced by inoculation type (**Figure 1**). MCF and MI increased over time (30 and 90 DAS) in plants inoculated with CS1 and CS2 under both irrigation conditions evaluated (WW and WS). Interestingly, in this study at 90 DAS, MCF and MI were not influenced by WS in the two consortia evaluated. However, the MI of the fungus *F. mosseae* present in CS2 decreased drastically (by 87%) compared to the fungus *C. claroideum* present in CS1 at both 30 and 90 DAS under WW and WS conditions (**Figure 1**). The percentage obtained for MCF and MI in the control suggest that not all the spores of these fungi die during the soil autoclaving process. Accordingly, we recommend conducting two soil sterilization cycles specifically, with a 24-hour interval, to ensure the complete elimination of any infective propagules of native AMF. However, the low values obtained had no effect on other parameters evaluated in this study.

### 3.2 Nutrient concentration in strawberry leaves

The nutrient concentrations in strawberry leaves were affected by the water deficit (**Table 2**). The concentrations of N, K, Ca, and Mg decreased by up to 16.8%, 12%, 23.2%, and 25.3%, respectively, in the WS control compared to WW control. However, there were no statistically significant differences (*p >* 0.05) in Ca concentrations between inoculation type, irrigation condition, and their interaction (**Table 2**). Interestingly, under WS conditions, in plants co-inoculated with CS1, the concentrations of N, P, K, and Mg increased by 24.3%, 6.4%, 25.7%, and 18.2%, respectively, compared to the WS uninoculated control. In addition, this consortium outperformed CS2 in terms of nutrient concentrations under water deficit, and even K concentrations in CS1WS (9.83%) were statistically higher than those in the well-watered control (8.30%).

**Table 2:** Nitrogen (N), phosphorus (P), potassium (K), calcium (Ca), and magnesium (Mg) concentration in strawberry leaves after inoculation with microbial consortia under well-watered (irrigated to 85% water holding capacity (WHC)) and water deficit (irrigated to 30% WHC) conditions.

Treatments	Irrigation condition	N (%)	P (%)	K (%)	Ca (%)	Mg (%)
Control	WW	1.31 ± 0.06ab	0.30 ± 0.01ab	8.30 ± 0.44d	2.50 ± 0.27a	1.82 ± 0.08a
CS1	WW	1.15 ± 0.05cd	0.31 ± 0.00a	10.60 ± 0.26a	2.25 ± 0.33a	1.58 ± 0.04ab
CS2	WW	1.26 ± 0.09bc	0.32 ± 0.01a	9.13 ± 0.04bc	2.32 ± 0.21a	1.72 ± 0.07ab
Control	WS	1.09 ± 0.04d	0.29 ± 0.00bc	7.30 ± 0.04e	1.92 ± 0.24a	1.39 ± 0.05b
CS1	WS	1.44 ± 0.04a	0.31 ± 0.00a	9.83 ± 0.23b	2.20 ± 0.03a	1.70 ± 0.05ab
CS2	WS	1.08 ± 0.04d	0.28 ± 0.00c	8.73 ± 0.37cd	2.20 ± 0.08a	1.42 ± 0.10b
Significance						
IN		*	**	***	ns	ns
IC		ns	*	***	ns	**
INxIC		***	***	*	ns	**

WW, well-watered, irrigated up to 85% water holding capacity (WHC); WS, water-stressed, irrigated up to 30% WHC. CS1, *C. claroideum* + *N. albida* + *P. caledonica*; CS2: *F. mosseae* + *C. guillermondii* + *B. tequilensis*; IN, inoculation type; IC, irrigation condition. The significant difference was depicted as ns, not significant, *p < 0.05, **p < 0.01, and ***p < 0.001. Values represent means ± SE. Different letters indicate significant differences using Tukey’s test (*p ≤* 0.05).

### 3.3 Profile of metabolites identified in strawberry leaves

**Figure 2:**
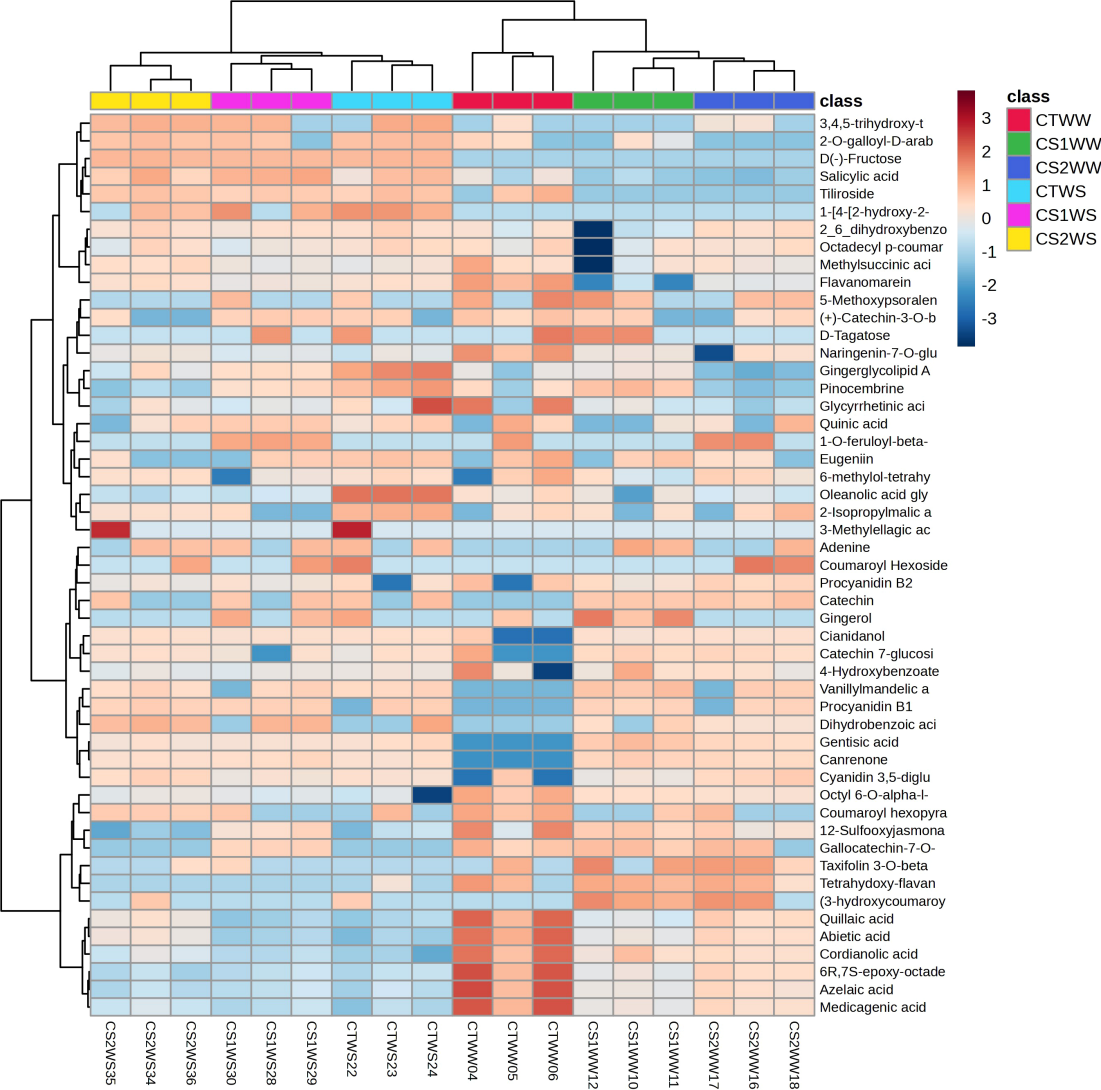
Heat map indicating the metabolite profile in strawberry plants inoculated with microbial consortia under two irrigation regimes. WW: well-watered (irrigated up to 85% WHC); WS: water-stressed (irrigated up to 30% of WHC). CS1: *Claroideoglomus claroideum* + *Naganishia albida* + *Paraburkholderia caledonica* CS2: *Funneliformis mosseae* + *Candida guillermondii* + *Bacillus tequilensis.*.

Untargeted metabolomics analysis was performed using ultra-high-performance liquid chromatography (UHPLC-MS/MS) and its variant, hydrophilic interaction liquid chromatography (HILIC-MS/MS), to identify changes in the primary and specialized metabolite profiles influenced by the evaluated irrigation conditions (WW and WS) and the microbial consortium. A heat map was created to identify similarities and differences between the evaluated irrigation conditions based on the relative concentrations of metabolites found in the different treatments. This clearly separated the well-irrigated treatments from the stressed ones (**Figure 2**). Compared to the well-watered group, an increase in the relative concentrations of salicylic acid, D(-)-fructose, 1-[4-[2-hydroxy-2-(4-hydroxy-3-methoxy-phenyl)-1-methylol-ethoxy]-3-methoxy-phenyl]-2-(4-hydroxy-3-methoxy-phenyl)propane-1,3-diol, 3,4,5-trihydroxy-tetrahydropyran-2-carboxylic acid, and 2-O-galloyl-D-arabino-pentopyranose was observed in all treatments under water-deficit stress. Likewise, the water deficit induced a decrease in the concentrations of arjunolic acid, octyl 6-O-alpha-l-arabinopyranosyl-beta-d-glucopyranoside, 12-oxo-PDA, Cordianolic acid, 6R,7S-epoxy-octadecanoic acid, azelaic acid, medicagenic acid, suberic acid, 5-Methoxypsoralen, and Tetrahydoxy-flavanone pentoside compared with the well-water. The concentrations of oleanolic acid glycoside, eriodictyol and gallic acid increased in stressed control plants, however, plants inoculated with microbial consortia under the same irrigation (WS) conditions showed a decrease in these metabolites (**Figure 2**). In this study, we observed differential modulation of metabolites in response to water deficit following inoculation with different microbial consortia (CS1 and CS2). Plants inoculated with CS2 and control plants under stress exhibited decreased levels of the flavonol gallocatechin-7-O-gallate and the phytohormone 12-sulfoxyjasmonate, in contrast to plants inoculated with CS1, which exhibited increased concentrations of these two metabolites. However, CS2 increased the concentrations of the terpenoid quillaic acid and abietic acid under stress compared to CS1 and the control, where concentrations decreased. In contrast, a similar response was obtained in both consortia (CS and CS2) for the phenolic gallic acid compound, where its concentration decreased in both consortia compared to the control, which increased under stress (**Figure 2**). The differential responses observed in plants inoculated with different consortia compared to control plants enabled us to identify the functional compatibility of these microorganisms, which varies depending on the plant species and type of stress.

### 3.4 Identification of significantly altered compounds in strawberry leaves

Multivariate analysis of the data using principal component analysis (PCA) enabled the distinction between the well-irrigated and stressed treatment groups. PCA explained 42.5% of the variability; most variables were associated with PC1 (25.8% variance), whereas PC2 explained a smaller proportion (16.7%) (Supplementary Materials **Supplementary Figure 1**). However, partial least squares discriminant analysis (PLS-DA) was used to identify significant changes in metabolites between groups (**Supplementary Figure 2**). PLS-DA allowed the identification of key metabolites between the different consortia and the control treatment using variable importance in projection (VIP).

**Figure 3:**
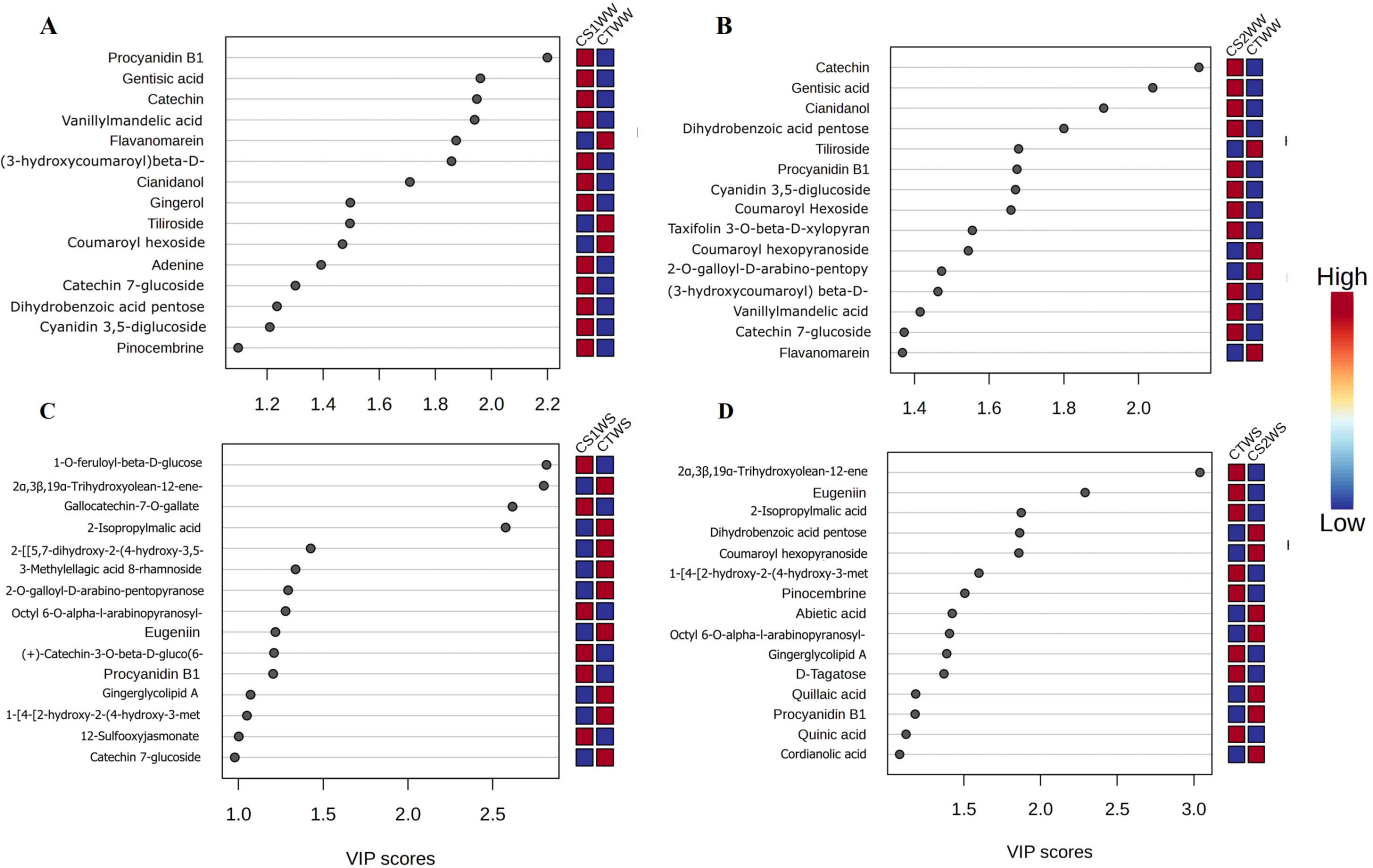
VIP scores from PLS-DA analysis showing the discriminant metabolites leading to the separation between control and microbial consortia (CS1 and CS2) under well-watered (WW) **(A, B)** and stressed (WS) **(C, D)** conditions. The colored boxes on the right indicate the relative concentrations of the corresponding metabolites. Red indicates high and blue indicates low. WW, well-watered, irrigated up to 85% WHC; WS: water-stressed irrigated up to 30% WHC; CT, control; CS1, *Claroideoglomus claroideum* + *Naganishia albida* + *Paraburkholderia caledonica*. CS2, *Funneliformis mosseae* + *Candida guillermondii* + *Bacillus tequilensis.*

The differentially accumulated metabolites against the control (CT) with a VIP score > 1 under non-stress conditions (WW) are shown in **Figures 3A, B**. In this respect, metabolites such as Gentisic acid, Vanillylmandelic acid, (3-hydroxycoumaroyl) beta-D-xylo-pentopyranoside, Cianidanol, Procyanidin B1, Dihydrobenzoic acid pentose, Cyanidin 3,5-diglucoside, Catechin, and Catechin 7-glucoside were accumulated in high concentrations in both microbial consortia (CS1 and CS2) under WW conditions (**Figures 3A, 3B**), while Coumaroyl hexoside, Tiliroside, and Flavanomerin showed a low concentration (CS1 and CS2) compared to CTWW. However, under well-watered conditions, CS1 produced high levels of the phenolic compounds Gingerol, Adenine nucleotide, and the flavonoid flavanomarein, which were exclusive to this treatment. Meanwhile, CS2 obtained higher concentrations of the flavonoid glycoside Taxifolin 3-O-β-D-xylopyranoside, as well as lower concentrations of the hydrolyzable tannin 2-O-galloyl-D-arabino-pentopyranose and flavonoid glycoside Coumaroyl hexopyranoside compared to the control under WW (**Figure 3B**). On the other hand, the response under stress conditions changed, with an increase in hydroxycinnamic acid glycosides 1-O-feruloyl-beta-D-glucose and flavonoid Gallocatechin-7-O-gallate in CS1 compared to the control (**Figure 3C**), as well as a decrease in the triterpene saponin Oleanolic acid glycoside and the hydroxy fatty acids 2-Isopropylmalic acid in this same consortium with respect to CTWS, where their accumulation was higher (**Figure 3C**), being these four metabolites the most relevant in the CS1/CT contrast under water deficit. On the other hand, in the CS2/CT contrast under water deficit, the metabolites 2α,3β,19α-Trihydroxyolean-12-ene-24,28-dioic acid 28-beta-D-glucopyranosyl, Eugeniin, 2-Isopropylylmalic acid were found to be more relevant and at a low concentration in CS2 with respect to the control, and Dihydrobenzoic acid pentose and Coumaroyl hexopyranoside were at a high concentration in this same consortium.

**Figure 4:**
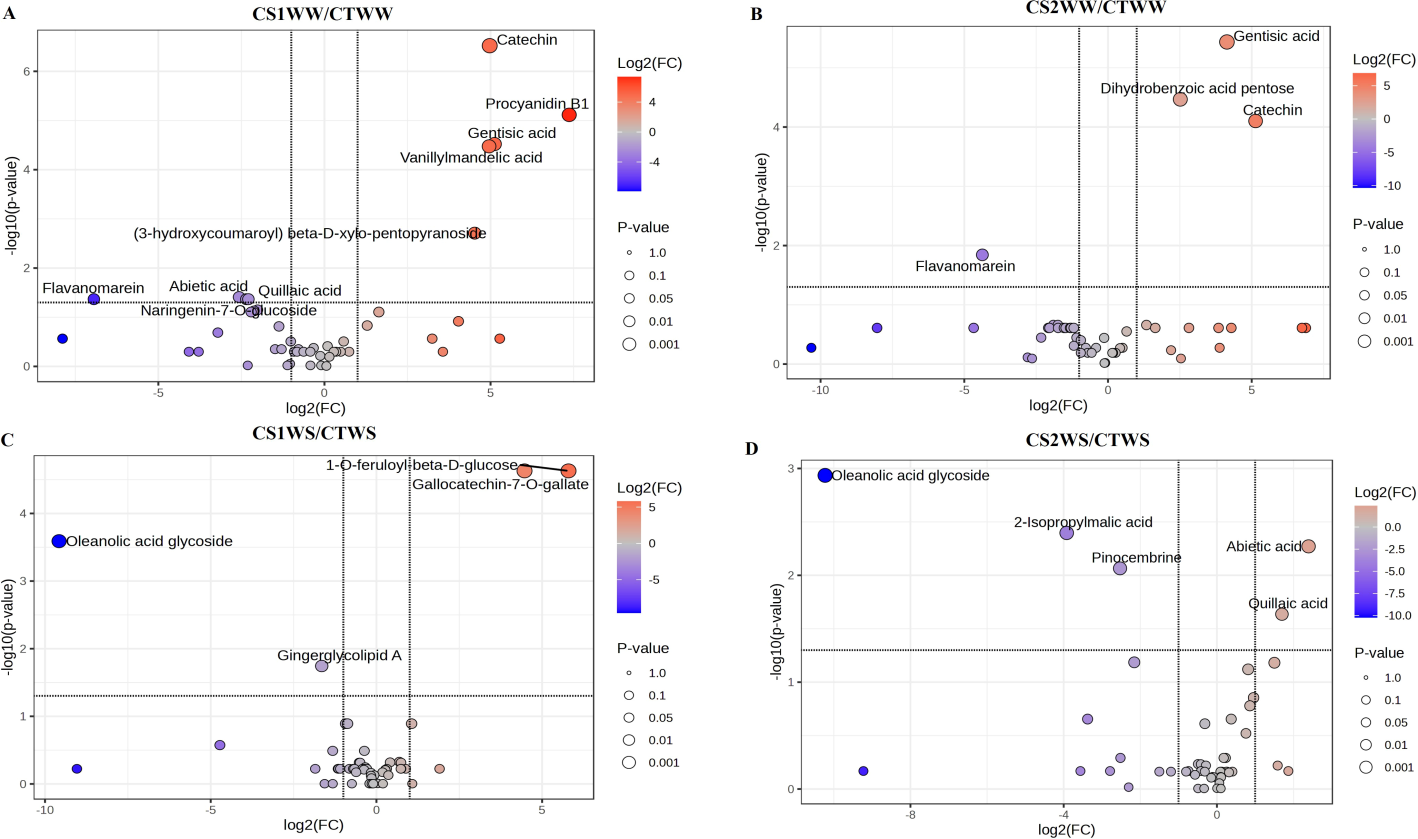
Significantly altered metabolites under stress (WS) and well-watered (WW) condition. Significant high concentrations metabolites are shown in red (log_2_FC > 2), significant low metabolites are shown in violet (log_2_FC < -2), and non-significantly different metabolites are shown in gray. The size of the dots represents the p-value (larger dot size represents a p<0.05). CT, control; CS1, *Claroideoglomus claroideum* + *Naganishia albida* + *Paraburkholderia caledonica*. CS2, *Funneliformis mosseae* + *Candida guillermondii* + *Bacillus tequilensis.*.

The volcano plot allowed the identification of metabolites that had a substantial change and were statistically significant (log_2_FC < -2 or log_2_FC > 2, p < 0.05 (FDR)) under the two water regimes. Under WW conditions, seven metabolites were found to be significantly altered in CS1, where they were mainly upregulated (**Table 3**; **Figure 4A**). Significant changes were observed in six metabolites in CS2, of which five had high concentration and one of which had a low concentration (**Table 3**; **Figure 4B**).

**Table 3:** Metabolites detected by LC-MS/MS and HILIC-MS/MS and tentatively annotated as differentially accumulated between inoculation treatments and control [log_2_FC < -2 or log_2_FC > 2, p < 0.05 (FDR)].

Comparasion	Mass	Annotation	Analysis	Formula	Log_2_(FC)	*P*-value (FDR)	Regulation
CS1WW vs CTWW	289.073	Catechin	HILIC-MS/MS	C_15_H_14_O_6_	5.21	1.04E-06	Up
	153.020	Gentisic acid	HILIC-MS/MS	C_7_H_6_O_4_	5.34	1.1E-05	Up
	577.138	Procyanidin B1	HILIC-MS/MS	C_30_H_26_O_12_	7.62	2.0E-05	Up
	197.047	Vanillylmandelic acid	HILIC-MS/MS	C_9_H_10_O_5_	5.22	1.0E-04	Up
	311.079	(3-hydroxycoumaroyl) beta-D-xylo-pentopyranoside	HILIC-MS/MS	C_14_H_16_O_8_	4.75	1.6E-03	Up
	255.067	Pinocembrine	LC-MS/MS	C_15_H_12_O_4_	2.56	1.1E-02	Up
	676.367	Gingerglycolipid A	LC-MS/MS	C_33_H_56_O_14_	2.25	1.6E-02	Up
CS2WW vs CTWW	153.020	Gentisic acid	HILIC-MS/MS	C_7_H_6_O_4_	4.21	2.1E-06	Up
	289.073	Catechin	HILIC-MS/MS	C_15_H_14_O_6_	5.2	7.6E-05	Up
	285.0617	Dihydrobenzoic acid pentose	HILIC-MS/MS	C_12_H_14_O_8_	2.58	3.6E-05	Up
	153.019	2-6-dihydroxybenzoic acid	HILIC-MS/MS	C_7_H_6_O_4_	2.15	2.0E-02	Up
	449.110	Flavanomerin	HILIC-MS/MS	C_21_H_22_O_11_	-3.53	8.0E-03	Down
CS1WS vs CTWS	355.104	1-O-feruloyl-beta-D-glucose	HILIC-MS/MS	C_16_H_20_O_9_	6.25	1.8E-05	Up
	491.086	Gallocatechin-7-O-gallate	HILIC-MS/MS	C_22_H_20_O_13_	4.92	7.1E-06	Up
	679.376	Oleanolic acid glycoside	LC-MS/MS	C_36_H_56_O_12_	-9.71	8.3E-04	Down
			
CS2WS vs CTWS	301.217	Abietic acid	LC-MS/MS	C_20_H_30_O_2_	2.32	6.1E-03	Up
	679.376	Oleanolic acid glycoside	LC-MS/MS	C_36_H_56_O_12_	-10.29	1.5E-03	Down
			
	255.067	Pinocembrine	LC-MS/MS	C_15_H_12_O_4_	-2.58	3.4E-03	Down
	175.061	2-Isopropylmalic acid	HILIC-MS/MS	C_7_H_12_O_5_	-3.97	1.5E-03	Down

A positive fold change (FC) value indicates that the metabolite in question is significantly higher in CS1 or CS2 than in the control. Conversely, a negative FC value indicates that the metabolite in question is significantly lower than the control. CT, control; CS1, *Claroideoglomus claroideum* + *Naganishia albida* + *Paraburkholderia caledonica*. CS2, *Funneliformis mosseae* + *Candida guillermondii* + *Bacillus tequilensi.s*

Under water deficit in the CS1/CT contrast three metabolites were found significantly altered within these two were up-regulated (Gallocatechin-7-O-gallate and 1-O-feruloyl-beta-D-glucose) and one down-regulated (Oleanolic acid glycoside) in CS1 with respect to the control (**Table 3**). In contrast, in the CS2/CT contrast, four metabolites were significantly altered, three of which were downregulated (Oleanolic acid glycoside, 2-Isopropylmalic acid and pinocembrin) and only one abietic acid was up-regulated in CS2. Based on these results, the water deficit significantly increased the concentration of triterpene saponin 2α,3β,19α-trihydroxyolean-12-ene-24,28-dioic acid 28-β-D-glucopyranoside in control plants. This compound was found to be the most significantly downregulated in both the CS1WS/CTWS and CS2WS/CTWS contrasts (**Figures 4C, D**; **Table 3**).

### 3.5 Metabolic pathways and set enrichment analysis of significantly altered compounds in strawberry leaves

**Figure 5:**
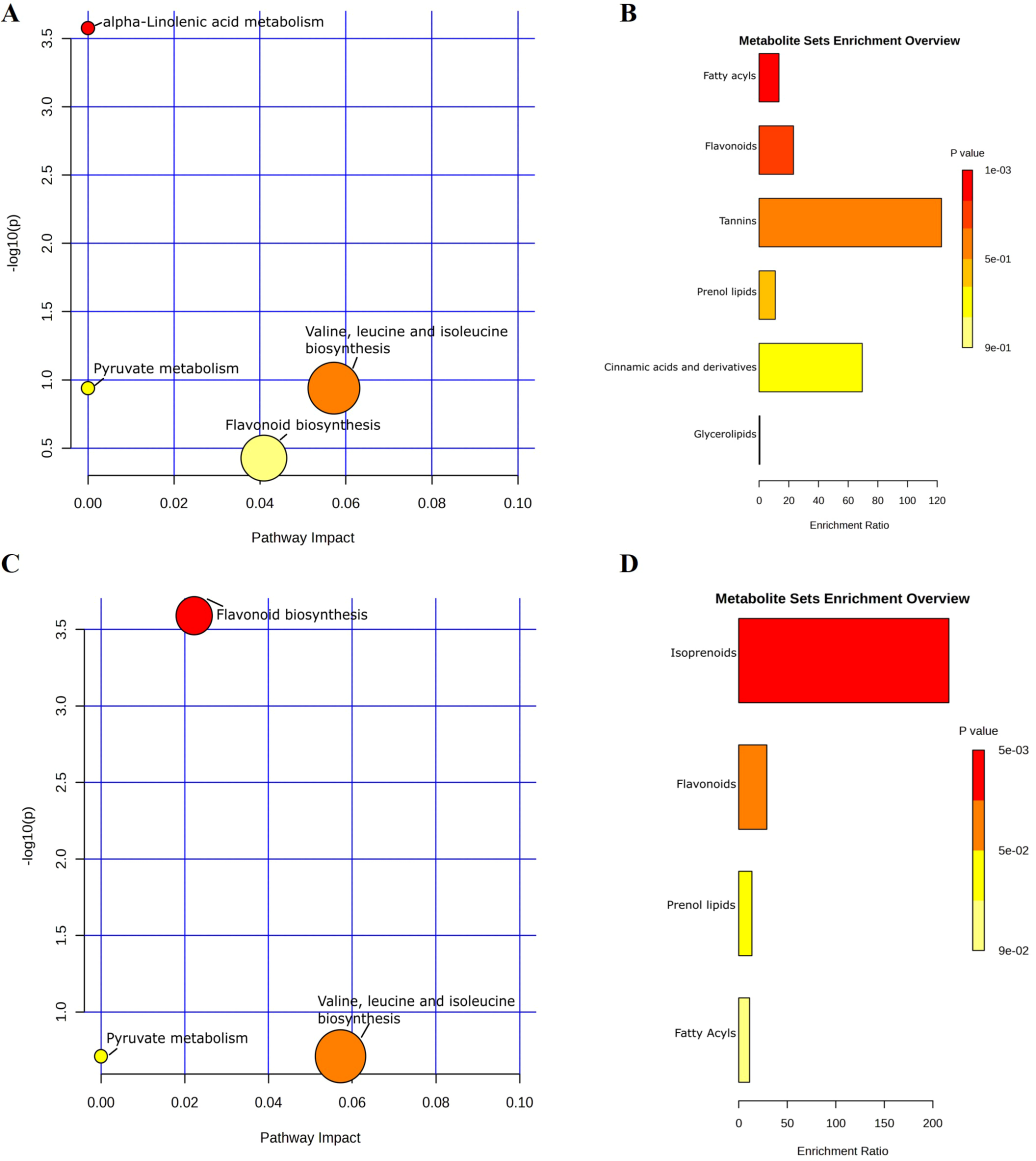
Metabolic Pathway Analysis and KEGG enrichment analysis of different treatment groups of strawberry plants under drought stress conditions after inoculation with two microbial consortia using MetaboAnalyst 6.0. Bubble size indicates the number of differentially altered metabolites associated with that specific pathway, and color intensity reflects statistical significance (*p* < 0.05). **(A, B)** CS1WS/CTWS contrast; **(C, D)** CS2WS/CTWS contrast.

The differential metabolites of plants inoculated with CS1 under drought stress compared to the non-inoculated control were mainly noted in four metabolic pathways. According to the bubble diagram, there were changes in the metabolites of alpha-linolenic acid metabolism, flavonoid biosynthesis, pyruvate metabolism y valine, leucine and isoleucine biosynthesis (**Figure 5A**). Similarly, CS2 under drought stress also produced changes in the metabolites of flavonoid biosynthesis metabolism, pyruvate metabolism, and valine, leucine, and isoleucine biosynthesis (**Figure 5C**). Although both consortia evaluated participated in the same metabolic pathways, such as flavonoid biosynthesis, pyruvate metabolism, and valine, leucine, and isoleucine biosynthesis, the most significant pathway for the CS1/CTWS contrast was alpha-linolenic acid metabolism, and for the CS2/CT contrast, it was flavonoid biosynthesis.

On the other hand, fatty acyls and flavonoid metabolism were the two most significant enrichment pathways for the CS1/CT contrast (**Figure 5B**), while for the CS2/CT contrast, the isoprenoids and flavonoids pathways were the most significant (**Figure 5D**). Both evaluated consortia were involved in the same metabolic pathways of flavonoids, with the difference that each consortium induced different metabolites (Gallocatechin-7-O-gallate in CS1 and Pinocembrine in CS2).

## 4 Discussion

### 4.1 Responses of strawberry plants under water shortage

The low availability of water to strawberry plants evaluated in this study caused changes at the morpho-physiological and phenotypic levels. The reduction in leaf and root biomass production demonstrated that this plant species is susceptible to water deficits, as reported in previous studies (Klamkowski and Treder, 2006; Zahedi et al., 2023; Thokchom and Hazarika, 2024). A study conducted by Liu et al. (2024) on strawberry plants (*Fragaria* × *ananassa* Duchesne) found that a severe water deficit reduced plant height, leaf dry biomass, and root dry biomass by up to 69.59%, 67.87%, and 79.92%, respectively. This reduction in plant biomass under low soil water availability can be attributed to a reduction in the relative water content of the cells, which results in reduced cell division, affecting all photosynthetic processes, and leads to slower growth (Liu et al., 2024). Additionally, the low biomass production of the plant may be due to the limited transport of nutrients from the soil to the plant owing to low water availability. As observed in our study, a water deficit reduced the leaf concentrations of elements such as N, K, and Mg in strawberry plants. Similar results were obtained by Partovi et al. (2025) in strawberry plants subjected to severe drought stress (30% field capacity (FC)). N and Ca uptake decreased by 28% and 34%, respectively, compared to well-watered plants (90% FC). It has been demonstrated that N, P and K play an important role in activating mechanisms in plants that help them to cope with stress. These mechanisms include carbohydrate accumulation, cell membrane stability, the regulation of enzymatic and non-enzymatic antioxidant defense mechanisms, the opening and closing of stomata to increase photosynthetic capacity, proline accumulation, and the prevention of reactive oxygen species accumulation (Kumari et al., 2022).

In contrast, the water deficit applied in our study remodeled the entire metabolome of the plant, activating metabolites from both primary and specialized metabolism. Under stress conditions, plants exhibited an increase in D(-)-fructose compared to plants under well-watered conditions (**Supplementary Figure 3**). Fructose, a type of soluble sugar, works alongside glucose and sucrose to trigger stress-related genes under abiotic stress conditions. These genes play a role in preserving the integrity of membranes and proteins, facilitating osmolite biosynthesis, regulating cell division and growth, and ensuring the balance of osmotic and ionic conditions (Sami et al., 2016; Jeandet et al., 2022). While the accumulation of carbohydrates, such as fructose, is typically associated with photosynthesis, our study indicates that the accumulation of fructose in stressed plants without inoculation of microbial biostimulants did not enhance stress tolerance. In contrast, we propose that it may have exerted an inhibitory effect on these processes. For instance, elevated sugar concentrations in plants have been shown to significantly inhibit photosynthetic processes, resulting in stunted growth (Paul et al., 2001; Moore et al., 2003; Rolland et al., 2006; Aubry et al., 2024). Meanwhile, the remodeling of specialized metabolism in well-watered plants compared to stressed plants without inoculation underwent a major change. While terpenoid metabolites such as abietic acid, quillaic acid, cordianolic acid, and medicagenic acid, as well as flavonoid metabolites such as gallocatechin-7-O-gallate and flavanomarein, responded to the increase in well-watered control, in the stressed control the relative concentrations of these metabolites decreased dramatically. Terpenoids are volatile plant metabolites that mediate communication between plants and other organisms and are biologically and ecologically relevant to plant defense against insects (Boncan et al., 2020). They are synthesized sequentially from isopentenyl diphosphate C5 (IPP) via mevalonic acid (MVA) within the cell (Liang et al., 2021). According to the number of carbon (C) atoms, they can be classified as 10 C monoterpenes, 15 C sesquiterpenes, 20 C diterpenes and 30 C triterpenes (Biswas and Dwivedi, 2019). In plants, triterpenes are found as free esters and conjugates of glycosides called saponins (Szakiel et al., 2011). These are high-molecular-weight glycosides consisting of a sugar moiety linked to a triterpene or steroid aglycone. Saponin precursors typically undergo several modifications before the addition of sugar molecules. Some studies have found that triterpenoid saponins increase under drought stress in plants (Nasrollahi et al., 2014; Yang et al., 2022; Chipkar et al., 2022), and many of them have antioxidant activity (Okubo and Yoshiki, 2000; Ghosh and Sil, 2013). However, in our study, the stress caused by limited irrigation did not cause an increase in these types of compounds, resulting in damage to the stressed plants.

### 4.2 Responses of strawberry plants to inoculation with microbial consortia under water scarcity

The potential of microbial consortia as biotechnological tools to cope with drought stress is being increasingly evaluated. Some studies have demonstrated that microbial consortia can have a more significant impact on plant drought tolerance than individual microorganism inoculation (Ashwin et al., 2022; Begum et al., 2022; Tyagi et al., 2023; Yim et al., 2025; Ahmad et al., 2025). This increase in tolerance has been demonstrated by increases in the aerial and root biomass of plants subjected to this stress, as well as an increase in the relative water content (RWC). For instance, Yim et al. (2025) discovered that inoculated maize seeds with individual bacterial isolates had no significant impact on the growth of plants. However, inoculation with the consortia produced significant increases in fresh and dry shoot mass, with increases of up to 41% (consortium 1) under drought conditions compared to non-inoculated controls. Another study on maize plants showed that inoculation with a microbial consortium consisting of *Serendipita indica*, *Rhizophagus intraradices*, and *Azotobacterchroococcum* increased the plants’ drought tolerance by increasing root and shoot length, root and shoot fresh weight, and root and shoot dry weight. This offered a greater advantage than applying these microorganisms individually (Tyagi et al., 2023). Although our study did not evaluate the individual effects of the different microorganisms inoculated in consortia (CS1 and CS2), CS1 exhibited statistically significant differences in shoot dry weight (SDW) under well-watered (WW) and water stress (WS) conditions compared to control and CS2-inoculated plants. These results are consistent with those obtained in previous studies (Pérez-Moncada et al., 2024), in which strawberry plants inoculated with CS1 under stress conditions showed increases in leaf dry biomass of up to 16.6% and in RWC of 12.4%, compared to stressed non-inoculated plants.

The increase in dry biomass found in strawberry plants inoculated with CS1 under water deficit conditions may be related to an increase in the mycorrhization intensity (MI) by the mycorrhizal fungus *Claroideglomus claroideum* present in this consortium (**Figure 1**), compared to the mycorrhizal fungus *Funneliformis mosseae* present in CS2. The increase in MI observed in this consortium (CS1) suggests that the simultaneous inoculation of the bacterium *P. caledonica* and the yeast *N. albida* promoted the colonization of AMF in strawberry plant roots. This statement is supported by Ech-Chatir et al. (2025), who reported that inoculation with a bacterial consortium increased mycorrhizal colonization by up to 100% in maize plants under drought conditions, in contrast to plants treated solely with the mycorrhizal consortium. Likewise, El-Beltagi et al. (2025) found that joint inoculation of *Bacillus amyloliquefaciens* and *Funneliformis mosseae* resulted in improved mycorrhizal colonization more than single inoculation of the mycorrhizal fungus. This led to significant improvements in plant height, leaf area and dry weight (El-Beltagi et al., 2025). On the other hand, our study observed statistically significant differences obtained in mycorrhization intensity (MI). MI was higher in plants co-inoculated with *C*. *claroideum*, *N. albida*, and *P. caledonica* than in plants co-inoculated with *F. mosseae*, *C. guillermondii*, and *B. tequilensis* in strawberry plants subjected to water deficit. These results suggest that the degree of colonization by AMF depends on the co-inoculated microorganisms, as observed in other studies (Nader et al., 2024; Mondlhane et al., 2026).

The increase in MI and biomass in plants inoculated with CS1 under water deficit conditions suggests that the combination of introduced mycorrhizal fungi (*C. claroideum*), bacteria (*P. caledonica*) and yeast (*N. albida*) can improve the ability of strawberry plants to absorb soil nutrients, particularly N and P, during drought. A previous study showed that this microbial consortium increased the N and P concentrations in strawberry plants subjected to water deficit by 6.6% and 11.7%, respectively, compared to the uninoculated water-stressed (WS) control (Pérez-Moncada et al., 2024). These increases in nutrient concentration can be attributed in part to the fact that yeasts possess mechanisms, such as the production of organic acids and secretion of enzymes (Bilek et al., 2020; Alzandi and Naguib, 2022), that facilitate the solubilization of P, thereby making it accessible to plants. A notable example is the yeast *Naganisha albida*, which has been isolated from extreme environments and demonstrates the ability to solubilize P (Raklami et al., 2024; Carrillo et al., 2025). It is an essential macroelement for plants and plays an important role in growth. However, drought stress can affect its availability in the soil, exacerbating its sorption owing to limited water availability under these conditions. This leads to reduced mobilization and uptake by the plant (Khan T. A. et al., 2024; Mahmood et al., 2025). On the other hand, plant growth-promoting bacteria can solubilize P, K and fix N from air. This is exemplified by the *Paraburkholderia* genus, which has plant growth-promoting characteristics (Sawana et al., 2014). An example of this is *Paraburkholderia caledonica*, which was evaluated in this study. This genus of bacteria has the potential to increase crop yields when used as a biofertilizer (Bellés-Sancho et al., 2023; Rojas-Rojas et al., 2025). The potential release from soil matrix of P by yeast, along with the release of K and N fixation by the bacterium, together with the efficient nutrient uptake through the external mycelium network facilitated by the AMF constituting the CS1 evaluated in this study, enabled strawberry plants to enhance their tolerance to water stress. A study by Nader et al. (2024) found that the presence of drought-tolerant bacteria producing indole acetic acid (IAA), gibberellic acid (GA), exopolysaccharides (EPS), and P solubilisers, together with AMF, increased the levels of N, P, and K in soybean plants under drought stress compared to untreated plants. Sheteiwy et al. (2021) investigated the impact of inoculating soybean plants with *Bacillus amyloliquefaciens* and a consortium of AMF, including *Acaulospora laevis*, *Septoglomus deserticola*, and *Rhizophagus irregularis*, under two distinct irrigation conditions: well-irrigated and drought-stressed environments. They found that co-inoculation with *B. amyloliquefaciens* and AMF resulted in the highest leaf N concentration. Evidence from different studies has shown that N deficiency alters photosynthetic membrane lipids, such as monogalactosyldiacyglycerol (MGDG) and digalactosyldiacylglycerol (DGDG), which are crucial for sustaining chloroplast structure and function (Liu et al., 2020; Peng et al., 2023; Li et al., 2024). This was demonstrated in previous studies where the CS2 evaluated in this study showed low accumulation of photosynthetic membrane lipids such as MGDG, SQDG, and DGDG (Pérez-Moncada et al., 2026), which we suggest may have occurred in response to low N concentration in the leaves of strawberry plants inoculated with this consortium.

Furthermore, research has shown that inoculation of PGPM in consortia can influence the synthesis of both primary and specialized metabolites in plants, thereby enhancing their tolerance to environmental stress (Abou Jaoudé et al., 2025a, Abou Jaoudé et al., 2025b; Francioli et al., 2025). In our study, water deficit led to an increase in the levels of salicylic acid (SA), a plant growth hormone, with its relative concentration being higher in CS1 than in CS2 and control plants (**Figure 2**; **Supplementary Figure 3**). SA plays a crucial role in enhancing plant stress tolerance by functioning as a signaling molecule and potent antioxidant that mitigates reactive oxygen species (ROS). It induces the expression of antioxidant enzymes in plants and facilitates the accumulation of proline and soluble sugars in plants (Song et al., 2023; Ikram et al., 2025; Thakur et al., 2025). Soluble sugars, such as D(-)-fructose, were found at high concentrations under water deficit in all evaluated treatments, although there were no significant differences between treatments under this stress. In contrast, water deficit greatly decreased the concentration of fatty acyls (octyl 6-O-alpha-l-arabinopyranosyl-beta-d-glucopyranoside, 12-oxo-PDA, 6R,7S-epoxy-octadecanoic acid, azelaic acid, suberic acid) in all treatments (**Figure 2**). Fatty acyls, which include fatty acids (FA) and their conjugates, are integral components of lipids (Labuda et al., 2018). Similar to our findings, drought stress has been shown to significantly reduce fatty acid composition in wheat (Ullah et al., 2022), sunflowers (Ghaffari et al., 2023), and maize plants (Yin et al., 2024). However, inoculation with microbial consortia can remodel fatty acid production under drought conditions, enabling plants to tolerate stress more effectively. Igiehon et al. (2021) identified several fatty acids with significant health benefits in soybean seeds when the plants were co-inoculated with *Rhizobium* spp. and mycorrhizal fungi under drought stress conditions. Similarly, the co-inoculation of P solubilizing bacteria (*Pantoea agglomerans* and *Pseudomonas putida*) with the arbuscular mycorrhizal fungus *Rhizophagus intraradices* in *Carum copticum* L. plants resulted in an increased percentage of petroselinic [64.28-70.11%], oleic [16.05-21.9%], and palmitic [6.50-9.10%] fatty acids under moderate water stress conditions, compared to non-inoculated control plants (Rezaei-Chiyaneh et al., 2021).

On the other hand, the fatty acyl pathway was activated (*p-value* = 0.00122) in the CS1/CT contrast under WS (**Figure 5B**), with the fatty acid conjugates 2-isopropylmalic acid and 12-sulfoxyjasmonate as the most enriched metabolic nodes in this pathway. 2-Isopropylmalic acid, an intermediate in the biosynthesis of the essential amino acid leucine in plants, has demonstrated antioxidant properties. However, its concentration decreased in both consortia under stress conditions compared to the control (**Supplementary Figure 3**). In contrast, the concentration of 12-sulfoxyjasmonate under stress increased in CS1 compared to CS2 and control plants (**Figure 3C**; **Supplementary Figure 3C**). 12-sulfoxyjasmonate is commonly associated with jasmonic acid (JA), a phytohormone derived from alpha-linolenic acid metabolism (Ruiz-Sanchez et al., 2011), which was significantly altered in this study (**Figure 5A**). Stomatal closure constitutes a fundamental mechanism of drought resistance, effectively mitigates water loss. JA operates upstream of abscisic acid (ABA) to initiate early drought responses and facilitate ABA accumulation in plants. Consequently, JA plays a pivotal role in modulating plant responses to drought stress through its direct action and interaction with other phytohormonal signaling pathways (Thakur et al., 2025). Furthermore, JA has been found to have a positive effect on the regulation of antioxidant enzymes (Ruiz-Sanchez et al., 2011). For instance, the application of methyl jasmonate (MeJA) in alfalfa and ryegrass plants under drought stress reduced APX, POD, and CAT antioxidant activity compared to drought-stressed plants, accompanied by low levels of malonaldehyde (MDA) and superoxide anion (O_2_^-^) (Wei et al., 2025). On the other hand, the application of MeJA and the AMF *Funneliformis mosseae* on barley plants growing under drought conditions had a stronger antioxidant defense to reduce drought stress damage than AMF inoculation alone (Hamidian et al., 2023). Additionally, JA has been found to improve nutrient uptake in plants growing under drought stress (Khan T. A. et al., 2024), as observed in this study, where CS1 significantly increased (*p* < 0.05) the concentration of NPK in strawberry plant leaves.

Different metabolites were remodeled by microbial consortia in response to water deficit. The strongest response of significantly altered metabolites under WW conditions was higher in both contrasts (CS1 and CS2 vs. CT) (**Table 3**). Similarly, under WS conditions, CS1 exhibited a high response with two significantly metabolites: the flavonoid gallocatechin-7-O-gallate and the hydroxycinnamic acid glycoside 1-O-feruloyl-β-D-glucose. Gallocatechin-7-O-gallate is a type of catechin, a group of secondary or specialized metabolites in plants, synthesized through the flavonoid biosynthesis pathway, the second most activated pathway in the CS1/CT contrast (**Figure 5B**). Previous studies have indicated that the synthesis of this type of catechin is closely linked to environmental stress tolerance in plants (Cheng et al., 2022; Ahammed et al., 2023; Wang et al., 2024). Specifically, catechins, such as gallocatechins are reported to exhibit antioxidant properties and may promote photosynthesis by inhibiting ABA-induced stomatal closure (Sato et al., 2022; Ahammed et al., 2023). The application of (−)-catechin gallate and (−)-gallocatechin gallate, obtained from green tea leaves, suppressed ABA-induced stomatal closure in *Arabidopsis thaliana* plants subjected to drought stress, suggesting a potential role as chemical regulators of plant growth via Ca²^+^-signaling pathways under this stress (Sato et al., 2022). Although stomatal conductance was not measured in the present study, the accumulation of this metabolite in CS1 suggests a potential mechanism by which the consortium maintains plant physiology under water deficit conditions. In turn, 1-O-feruloyl-beta-D-glucose, a beta-D-glucoside resulting from the formal condensation of the carboxyl group of ferulic acid (https://pubchem.ncbi.nlm.nih.gov/compound/7196-71-6), is a phenolic acid commonly found in plant cell walls that provides structural support and protection against environmental stress (Khan K. A. et al., 2024). At adequate concentrations, ferulic acid has been shown to have positive effects, such as exhibiting antioxidant properties and increasing the activity of antioxidant enzymes, such as superoxide dismutase (SOD) and catalase (CAT), as well as regulating proline content. However, high concentrations of ferulic acid can have pro-oxidant effects, inhibit photosynthesis and cause phytotoxic effects in plants (Hussain and Reigosa, 2021; Linić et al., 2021; Khan K. A. et al., 2024). Our findings, combined with previous results where CS1 was shown to increase the activity of CAT and ascorbate peroxidase (APX) under WS conditions (Pérez-Moncada et al., 2026), support the hypothesis that the accumulation of 1-O-feruloyl-β-D-glucose in this study is part of a coordinated antioxidant response. This specialized metabolic reprogramming likely contributes to the enhanced tolerance observed in strawberry plants inoculated with the CS1 consortium. In contrast, the response of CS2 under WS remained low, with three significantly downregulated metabolites: the flavonone pinocembrin, 2-isopropylmalic acid, and the glycosidic triterpenoid oleanolic acid glycoside. The concentration and differential regulation of this glycosylated triterpenoid were common in CS1WS/CTWS and CS2WS/CTWS, in contrast to the relatively low concentrations found in CS1 and CS2, and the high concentration found in CT (**Figures 4C 4D,**). Although it has been found that terpenoid compounds increase with stress, giving the plant increased antioxidant activity, in our study, the triterpenoid saponin oleanolic acid glycoside was found in high concentrations (VIP > 1) and upregulated (log_2_FC < -2 or log_2_FC > 2, *p* < 0. 05 (FDR)) in the CTWS exhibited a deleterious biological activity as plant biomass and plant leaf nutrient contents were significantly decreased, compared to plants inoculated with CS1 under water deficit. Similarly, we propose that abietic acid, a diterpene that was enriched in the isoprenoid pathway in CS2 under water deficit and was the only metabolite to be significantly up-regulated (**Figure 4D**; **Table 3**), had a comparable impact to that of the triterpenoid oleanolic acid glycoside, which was a significantly up-regulated metabolite in control plants. Although no studies have directly linked abietic acid to drought stress, it has been found to play a role in plant defense against fungi and insects (Trapp and Croteau, 2001; Satish et al., 2020). In humans, it exhibits antibacterial, antifungal, antiviral, and antioxidant properties (Buommino et al., 2021; Hasan et al., 2024). As mentioned above, plants synthesize terpenoids via the mevalonate (MVA) pathway in the cytoplasm and the methyl erythritol 4-phosphate (MEP) pathway in plastids. P plays an important role in the MEP pathway as a component of glyceraldehyde phosphate and pyruvate. Therefore, P is an essential promoter of terpenoid synthesis (Lv et al., 2024). In our study, the control and CS2-inoculated plants had lower concentrations (*p* < 0.05) of this element than the CS1-inoculated plants under water stress (**Table 2**). This may have affected plant biomass owing to the phytotoxic effects of water deficit.

The findings of this study indicate that the application of microbial biostimulants, such as those assessed herein, influences the secondary or specialized metabolites of strawberry plants subjected to water-deficit conditions. This influence can be either beneficial or detrimental, depending on the specific microorganisms evaluated. For instance, both microbial consortia examined in this study shared a common pathway under water stress, namely flavonoid biosynthesis; however, their effects varied across the different treatment groups. While this pathway had a positive effect on plants inoculated with CS1 owing to significant gallocatechin-7-O-gallate accumulation, it had a negative effect on plants inoculated with CS2 owing to a significant decrease in pinocembrin (**Supplementary Figure 3**). Flavonoids are an important group of compounds in the phenylpropanoid pathway. They act as pigments, are soluble in water, and are stored in plant cell vacuoles (Al-Khayri et al., 2023). In particular, they act as antioxidants that inhibit ROS production during drought stress (Kaur and Suseela, 2020; Wang et al., 2025). Flavonoids also regulate the relationship between plants and microbes (Scervino et al., 2005; Weston and Mathesius, 2013; Wang et al., 2022, Wang et al., 2025). Francioli et al. (2025) conducted a comprehensive investigation into the effects of a microbial consortium, comprising *Pseudomonas* sp. RU47, *Bacillus atrophaeus* ABi03, and *Trichoderma harzianum* OMG16 on maize (*Zea mays* cv. Benedictio) through a long-term field experiment. This study incorporated drought periods as well as intensive and extensive agricultural practices. The researchers observed that plants inoculated with this consortium exhibited significant increases in flavonoid-type metabolites, including caffeic acid and quercetin/naringenin. Additionally, maize plants treated with a consortium of *S. indica*, *R. intraradices*, and *A.chroococcum* and subsequently exposed to drought conditions accumulated higher concentrations of phenols and flavonoids. This prevents the harmful effects of water stress on plants (Tyagi et al., 2023). Conversely, water deficit decreased the concentration of nutrients in the evaluated treatments, particularly N, P and K (**Table 2**). However, this decreases, particularly in N, promoted an increase in the flavonoid concentration in CS1 compared to CS2. Studies on N deficiency in plants have shown that this deficiency significantly increases the synthesis and secretion of flavonoids (Stewart et al., 2001; Li et al., 2024; Ninkuu et al., 2025), although this did not occur in plants inoculated with CS2.

## 5 Conclusions

This study highlights the critical role played by the specificity of microbial consortia in modulating the host plant’s resilience to water stress. Using untargeted LC-MS metabolomics, we have demonstrated that the efficacy of microbial biostimulants is highly specific, as different consortia uniquely reprogram the host’s secondary metabolism to activate protective antioxidant and hormonal signaling pathways—as observed in the consortium composed of *C. claroideum*, *N. albida*, and *P. caledonica*—or, conversely, fail to confer adaptive advantages. These contrasting results underscore that plant-microbe-environment interactions are highly specific, where microbial diversity dictates the host’s metabolic profile and subsequent stress tolerance. Ultimately, this work provides a fundamental framework for deciphering the molecular mechanisms of microbial biostimulant-mediated resilience and identifies specialized key metabolites as potential biomarkers of drought stress. To translate these findings into scalable agricultural practices, future long-term field studies are warranted to dissect the individual and synergistic contributions of these microbial strains, optimizing the formulation of targeted inoculants for sustainable crop production.

## Data Availability

The original contributions presented in the study are included in the article/**Supplementary Material**. Further inquiries can be directed to the corresponding authors.
