# Supplementary material for: Microbial consortia and their metabolic modulations as mechanisms of water deficit tolerance in strawberry plants
Source: Front Plant Sci. 2026 Jul 7;17:1843067. doi: 10.3389/fpls.2026.1843067 (PMC13384946; doi:10.3389/fpls.2026.1843067)
Supplement: Supplementary file 1 [file Table1.docx]

Supplementary Material

**
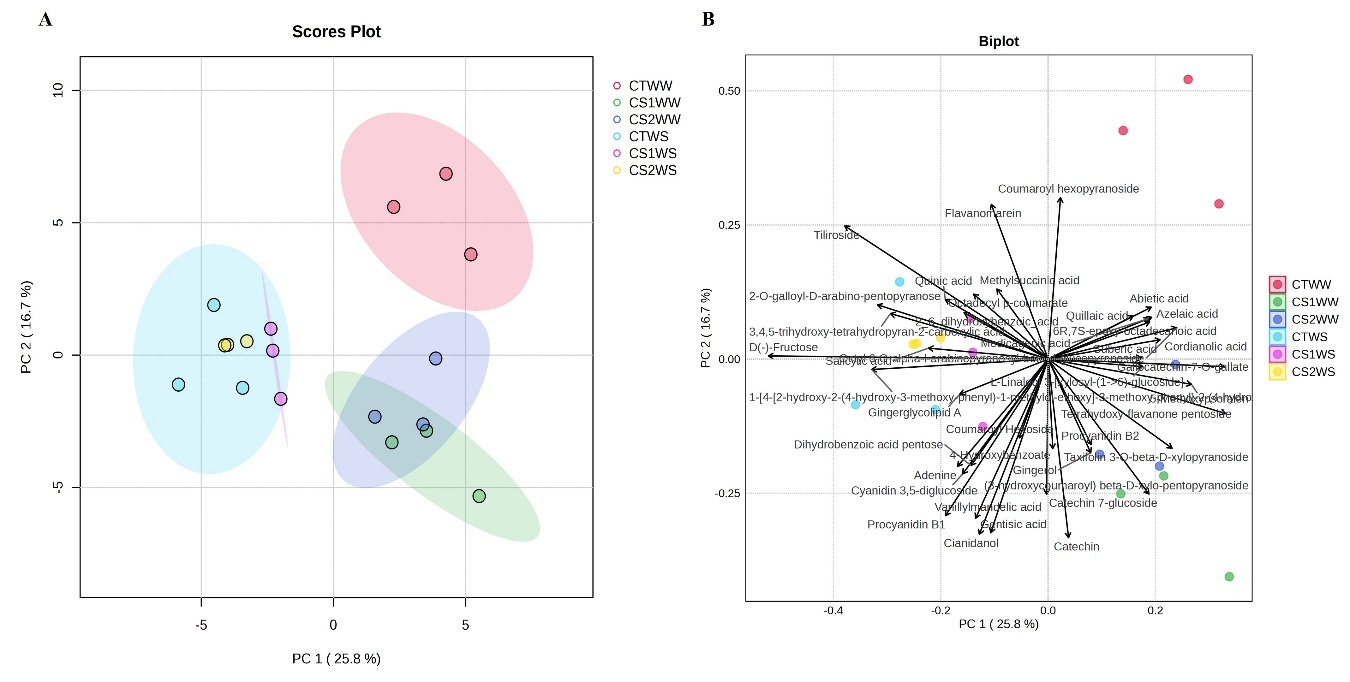
**

**Supplementary Figure S1.** Principal Component Analysis (A) and biplot (B) based on metabolites leading to the separation between control and microbial consortia (CS1 and CS2) under well-watered (WW) and stressed (WS) conditions. WW: well-watered, irrigated up to 85% WHC; WS: water-stressed irrigated up to 30% WHC; CT: control; CS1: *Claroideoglomus claroideum* + *Naganishia albida* + *Paraburkholderia cledonica* CS2: *Funneliformis mosseae* + *Candida guillermondii* + *Bacillus tequilensis.*


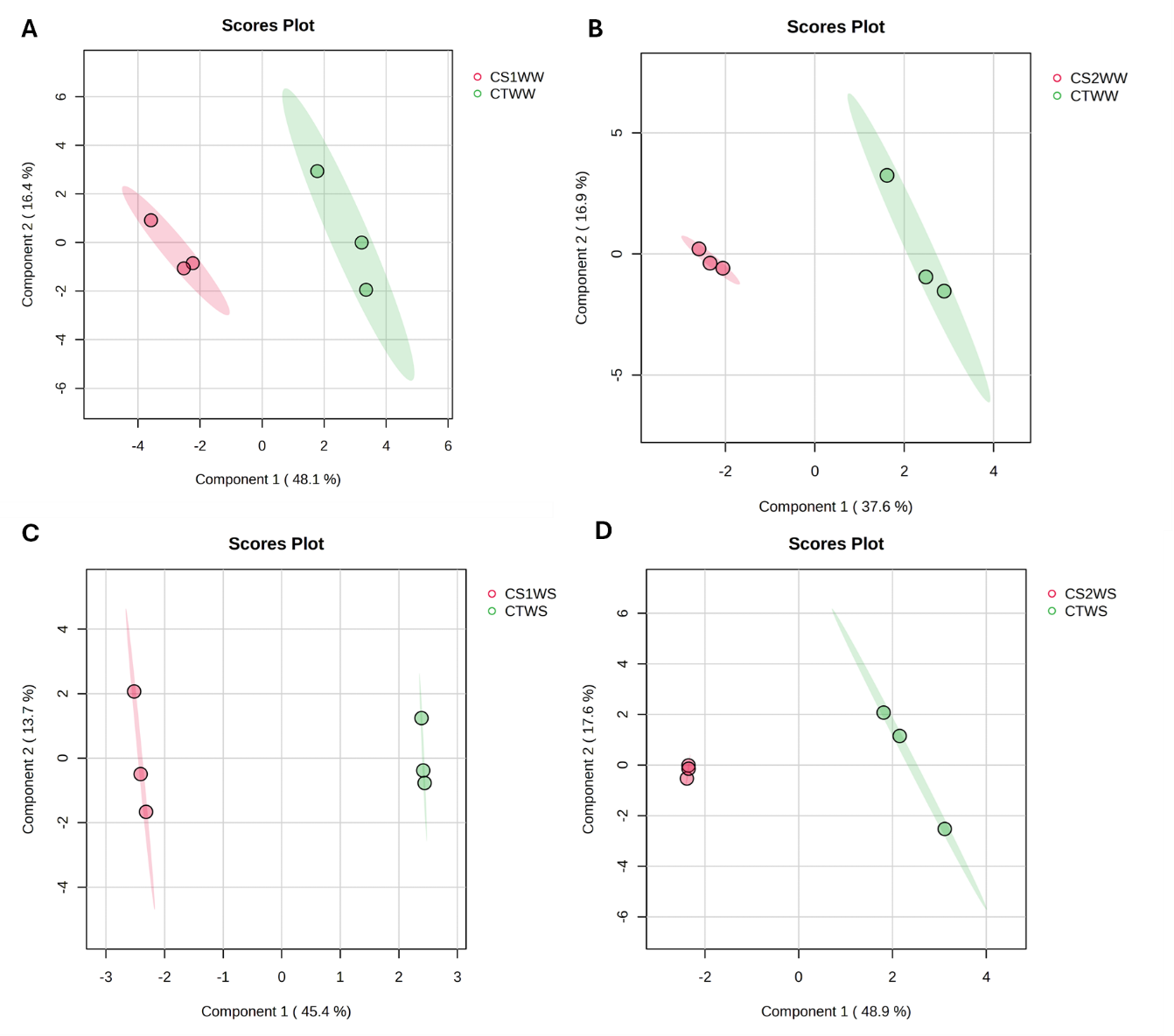


**Supplementary Figure S2.** PLS-DA analysis showing the discriminant metabolites leading to the separation between control and microbial consortia (CS1 and CS2) under well-watered (WW) (A and B) and stressed (WS) (C and D) conditions. WW: well-watered, irrigated up to 85% WHC; WS: water-stressed irrigated up to 30% WHC; CT: control; CS1: *Claroideoglomus claroideum* + *Naganishia albida* + *Paraburkholderia cledonica* CS2: *Funneliformis mosseae* + *Candida guillermondii* + *Bacillus tequilensis.*

**CTWW/CTWS**

*
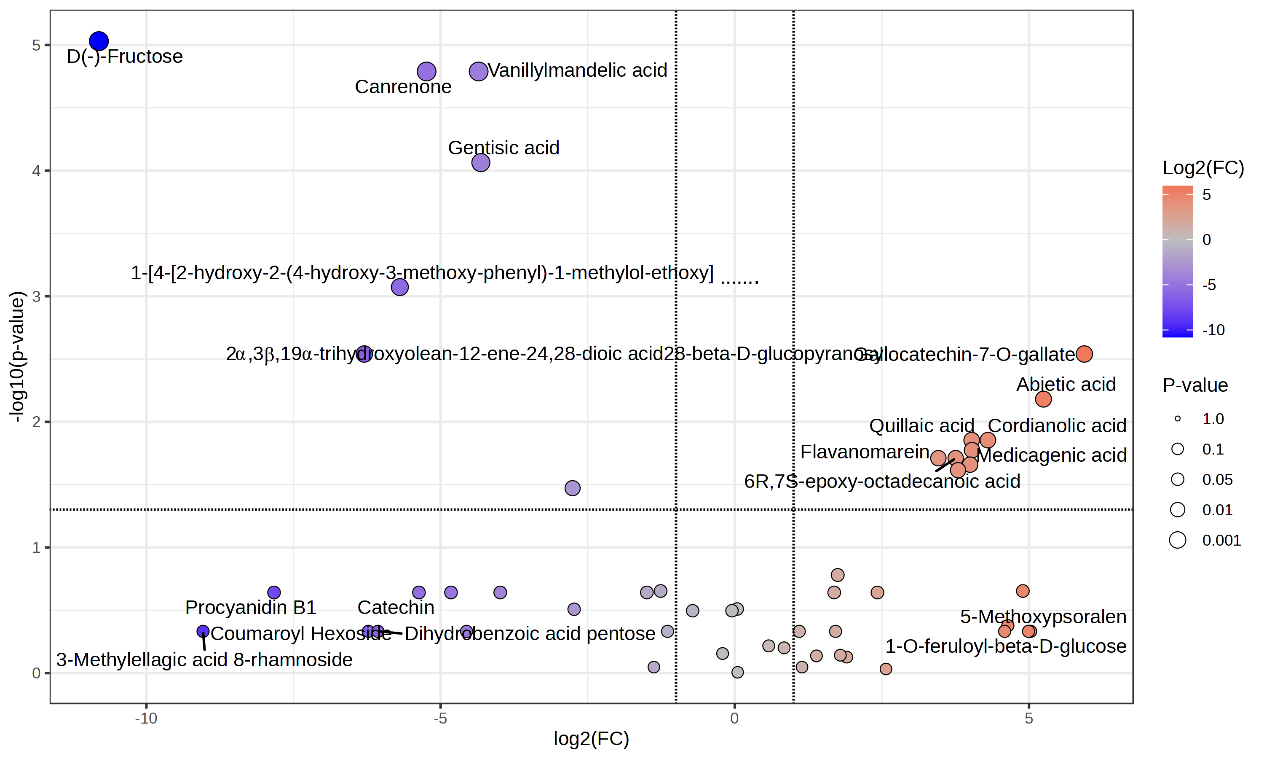
*

**Supplementary Figure S3.** Volcano plot of significantly altered metabolites under stress (WS) and well-watered (WW) condition. Significant up-regulated metabolites are shown in red (log_2_FC > 2), significant down-regulated metabolites are shown in violet (log_2_FC < -2), and non-significantly different metabolites are shown in gray. The size of the dots represents the p-value (larger dot size represents a *p<*0.05). CT: control.


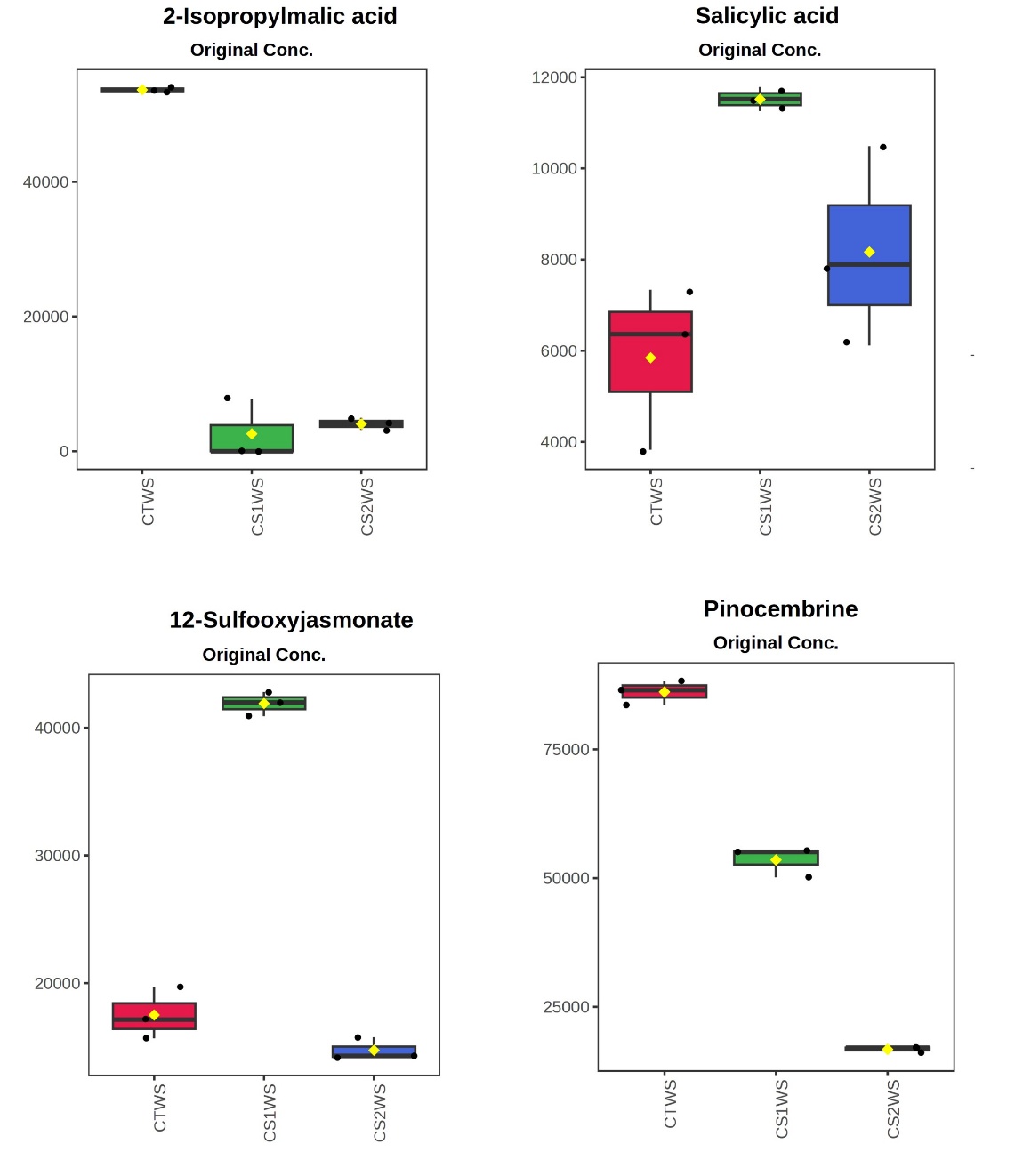


**Supplementary Figure S4.** Concentrations of metabolites specialized under WS conditions of strawberry plants inoculated with CS1 and CS2. Boxplot from VIP scores analysis. WS: water-stressed irrigated up to 30% WHC; CT: control; CS1: *Claroideoglomus claroideum* + *Naganishia albida* + *Paraburkholderia cledonica* CS2: *Funneliformis mosseae* + *Candida guillermondii* + *Bacillus tequilensis.*
